# CancerGPT for few shot drug pair synergy prediction using large pretrained language models

**DOI:** 10.1038/s41746-024-01024-9

**Published:** 2024-02-19

**Authors:** Tianhao Li, Sandesh Shetty, Advaith Kamath, Ajay Jaiswal, Xiaoqian Jiang, Ying Ding, Yejin Kim

**Affiliations:** 1 School of Information, University of Texas at Austin, Austin, TX USA; 2 Manning College of Information and Computer Sciences, University of Massachusetts Amherst, Amherst, MA USA; 3 Department of Chemical Engineering, University of Texas at Austin, Austin, TX USA; 4 McWilliams School of Biomedical Informatics, University of Texas Health Science Center at Houston, Houston, TX USA

**Keywords:** Machine learning, Virtual drug screening

## Abstract

Large language models (LLMs) have been shown to have significant potential in few-shot learning across various fields, even with minimal training data. However, their ability to generalize to unseen tasks in more complex fields, such as biology and medicine has yet to be fully evaluated. LLMs can offer a promising alternative approach for biological inference, particularly in cases where structured data and sample size are limited, by extracting prior knowledge from text corpora. Here we report our proposed few-shot learning approach, which uses LLMs to predict the synergy of drug pairs in rare tissues that lack structured data and features. Our experiments, which involved seven rare tissues from different cancer types, demonstrate that the LLM-based prediction model achieves significant accuracy with very few or zero samples. Our proposed model, the CancerGPT (with ~ 124M parameters), is comparable to the larger fine-tuned GPT-3 model (with ~ 175B parameters). Our research contributes to tackling drug pair synergy prediction in rare tissues with limited data, and also advancing the use of LLMs for biological and medical inference tasks.

## Introduction

Foundation models have become the latest generation of artificial intelligence (AI)^1^. Instead of designing AI models that solve specific tasks one at a time, such foundation models or “generalist” models can be applied to many downstream tasks without specific training. For example, large pre-trained language models (LLMs), such as GPT-3^2^ and GPT-4^3^, have been a game changer in foundation AI model^4^. An LLM can apply its skills to unfamiliar tasks for which it has never been trained, known as few-shot learning or zero-shot learning. This is due in part to multitask learning, which enables LLM to unintentionally gain knowledge from implicit tasks in its training corpus^5^. Although LLMs have shown proficiency in few-shot learning in various fields^2^, including natural language processing, robotics, and computer vision^2,6,7^, their generalizability to unseen tasks in more complex fields, such as biology, has yet to be fully tested. In order to infer unseen biological reactions, knowledge of participating entities (e.g., genes, cells) and underlying biological mechanisms (e.g., pathways, genetic background, cellular environment) is required. While structured databases encode only a small portion of this knowledge, the vast majority is stored in free-text literature, which can be used to train LLMs. Thus, we envision that when there are limited structured data and limited sample sizes, LLMs can serve as an innovative approach for biological prediction tasks, by extracting prior knowledge from unstructured literature. One of such few-shot biological prediction tasks with a pressing need is a drug pair synergy prediction in understudied cancer types.

**Fig. 1:**
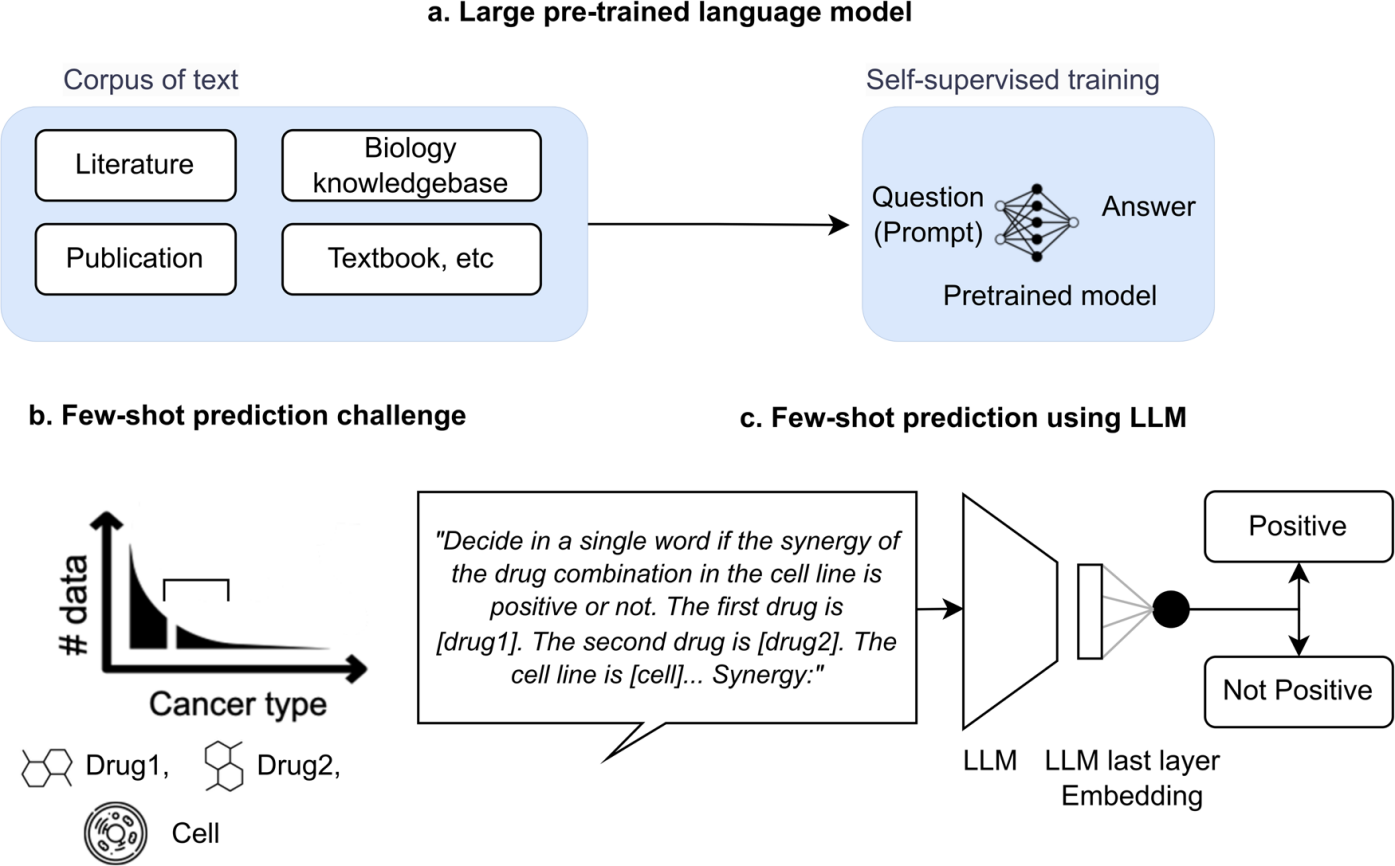
Few-shot prediction in biomedicine. **a** Different from a task-specific approach, a large pre-trained language model can perform new tasks for which it has not been explicitly trained. **b** Drug pair synergy prediction in rare tissues is an important example of the numerous few-shot prediction tasks in biomedicine. **c** A large pre-trained language model can be an innovative approach for few-shot prediction in biomedicine, thanks to the prior knowledge encoded in its weight.

Drug combination therapy has become a widely accepted strategy for treating complex diseases such as cancer, infectious diseases, and neurological disorders^8^. In many cases, combination therapy can provide better treatment outcomes than single-drug therapy. Predicting drug pair synergy has become an important area of research in drug discovery and development. Drug pair synergy refers to the enhancement of the therapeutic effects of two (or more) drugs when used together compared to when each drug is used alone. The prediction of drug pair synergy can be challenging due to a large number of possible combinations and the complexity of the underlying biological mechanisms^9^. Several computational methods have been developed to predict drug pair synergy, particularly using machine learning. Machine learning models can be trained on large datasets of existing drug pair’s experiment results to identify patterns and predict the likelihood of synergy for a new drug pair. Early studies in this area have relied on relational information or contextual information to extrapolate the synergy score regarding a specific cell line to cell lines in other tissues^10–14^, ignoring the biological and cellular differences in these tissues. Another line of studies has sought to overcome the discrepancy between tissues by utilizing diverse and high-dimensional features, including genomic (e.g., gene expression of cell lines) or chemical profiles (e.g., drug structure)^13,15–18^. However, most of the available data comes from common cancer types in certain tissues, such as breast and lung cancer; very limited experiment data are available on certain tissues, such as bone and soft tissue (Fig. 1). Obtaining cell lines from these tissues can be physically difficult and expensive, which limits the number of training data available for drug pair synergy prediction. This can make it challenging to train machine learning models that rely on large datasets. ref. ^18^ used transfer learning to extend the prediction model trained in common tissues to some of the rare tissues with relatively rich data and cellular features. However, it cannot be utilized for rare tissues with extremely limited data and cellular information.

In this work, we aim to overcome the above challenge by LLMs. We hypothesize that cancer types with limited structured data and features still have rich information in scientific literature. Manually extracting predictive information on such biological entities from literature is a complex task. Our innovative approach is to leverage prior knowledge in scientific literature encoded in LLMs. We build a few-shot drug pair synergy prediction model that leverages representation from LLM as a prior knowledge and predict the outcome. Our experimental results demonstrate that our LLM-based few-shot prediction model achieved significant accuracy even in zero-shot setting (i.e., no training data) and outperformed strong tabular prediction models in most cases. This remarkable few-shot prediction performance in one of the most challenging biological prediction tasks has a critical and timely implication to a broad community of biomedicine because it shows a strong promise in the “generalist” biomedical artificial intelligence^1^.

## Results

### Results overview

**Fig. 2:**
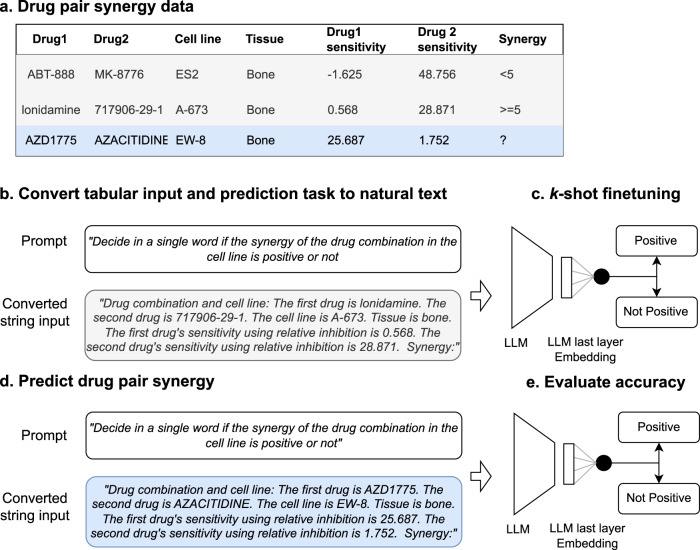
Study workflow. We converted the tabular input (**a**) to natural text and created a task-specific prompt (**b**). The prompt was designed for the LLM to give a binary answer (i.e., *“Positive”, “Not positive”*), and the LLM’s embedding from the last token was used for the final prediction (**c**, **d**). We evaluated and compared the prediction models with a different number of shots and tissues (**e**). We investigated the LLM’s reasoning based on factual evidence. LLM Large Language Model.

We developed CancerGPT, a few-shot drug pair synergy prediction model for rare tissues. Leveraging LLMs-based tabular data prediction model^19^, we converted the prediction task into a natural language task, derived embedding from prior knowledge encoded in LLM’s pre-trained weight matrices, and built classification model for synergy prediction (Fig. 2). We presented our strategy to adapt the LLM to our task with only a few shots of training data in each rare tissue using *k*-shot fine-tuning strategy (Fig. 6).

**Fig. 3:**
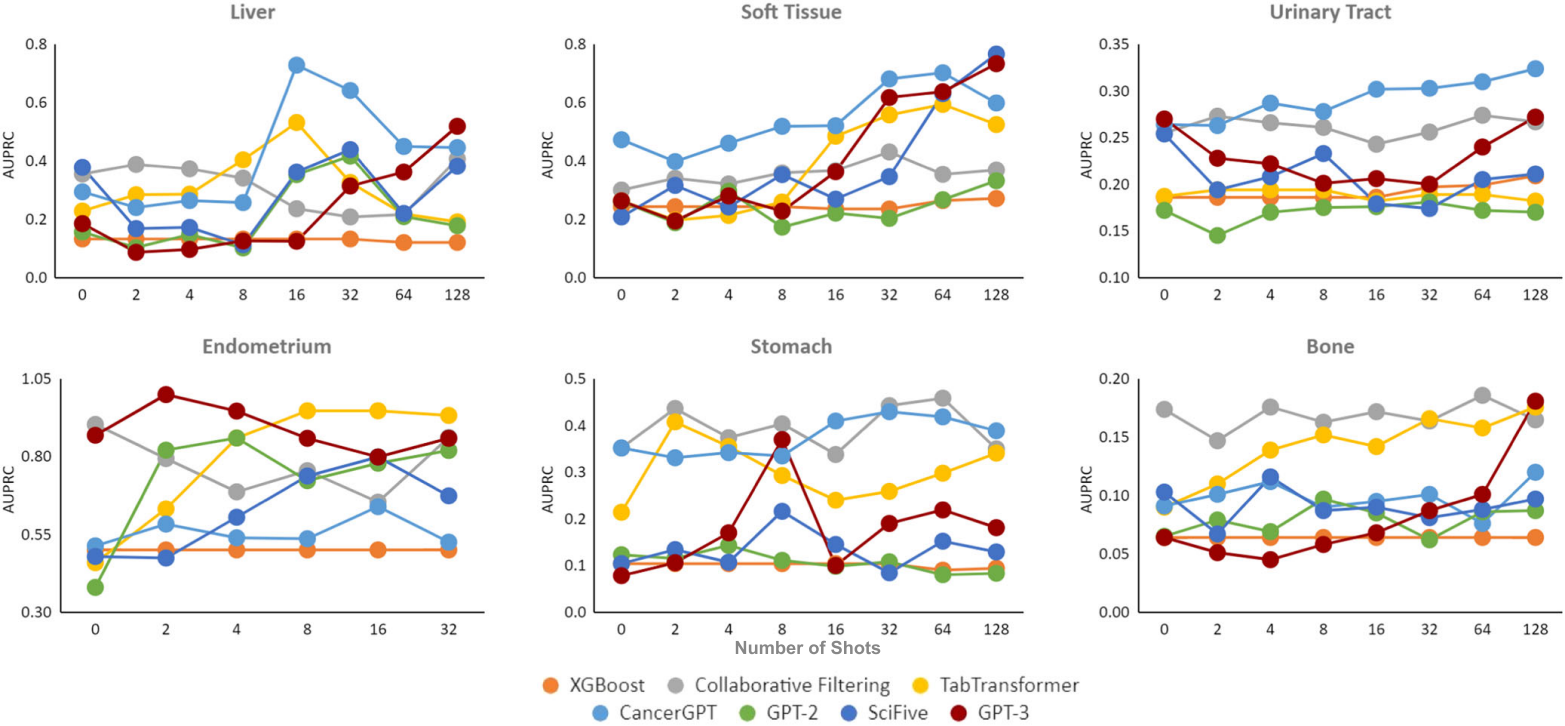
AUPRC of *k*-shot learning on seven tissue sets. We used 20% data as a test set in each rare tissue, while ensuring the binary labels were equally represented. AUPRC Area Under the Precision Recall Curve. Legend Orange: XGBoost, Gray: Collaborative Filtering, Yellow: TabTransformer, Light Blue: CancerGPT, Green: GPT-2, Dark Blue: SciFive, Red: GPT-3.

**Fig. 4:**
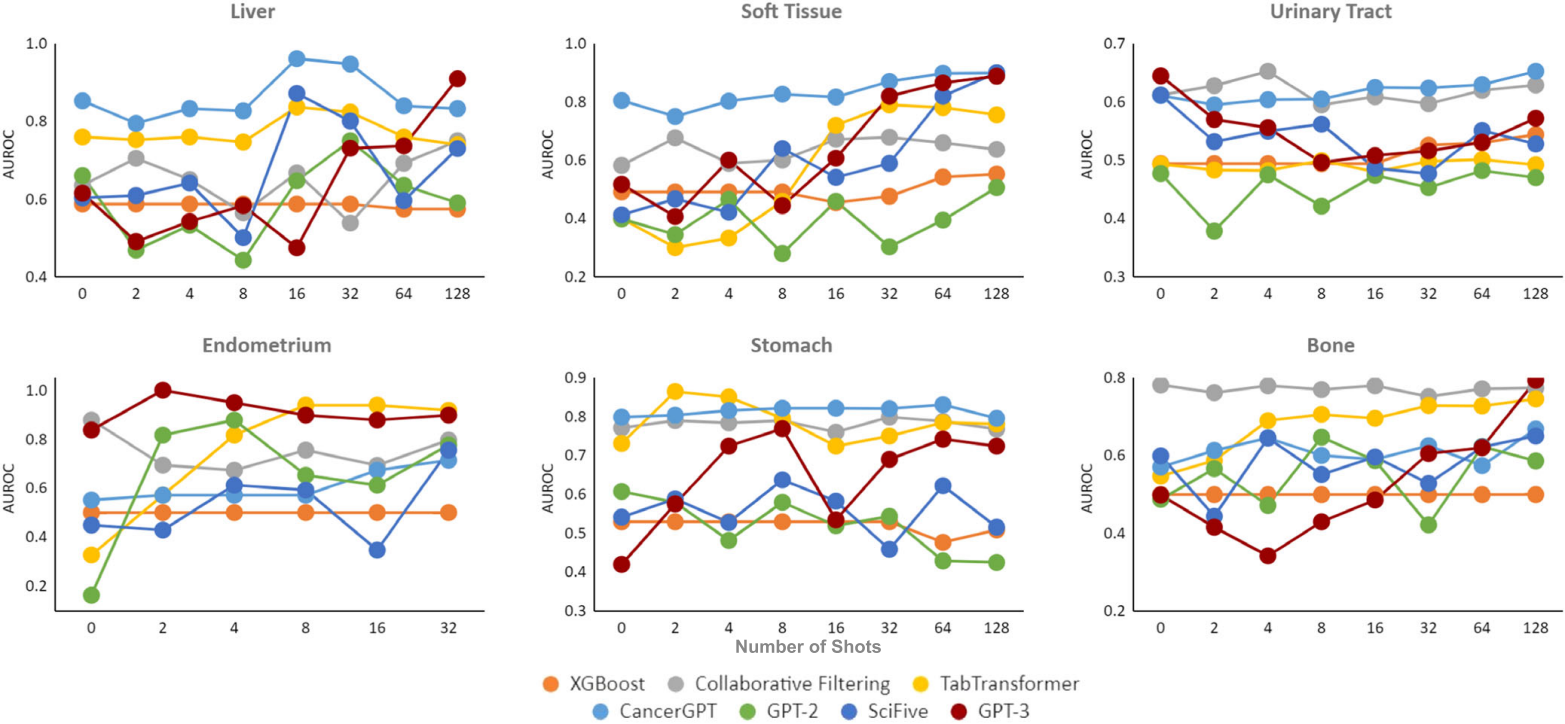
AUROC of *k*-shot learning on seven tissue sets. We used 20% data as a test set in each rare tissue, while ensuring the binary labels were equally represented. AUROC Area Under the Receiver Operating Curve. Legend Orange: XGBoost, Gray: Collaborative Filtering, Yellow: TabTransformer, Light Blue: CancerGPT, Green: GPT-2, Dark Blue: SciFive, Red: GPT-3.

We evaluated the accuracy of our proposed CancerGPT model, other LLM-based models (GPT-2, GPT-3, SciFive^20^), and general data-driven prediction models (XGBoost, Collaborative Filtering, TabTransformer) (Methods, Fig. 3, 4, Supplementary Figs. 1, and 2, Supplementary Table 2-5) by the area under the precision-recall curve (AUPRC) and the area under the receiver operating curve (AUROC) under the different settings. We considered different few-shot learning scenarios, where the model is provided with a limited number *k* of training data to learn from (*k* = 0 to 128). By varying the number of shots, we can examine the model’s ability to adapt and generalize with minimal training data. Next, we compared the performance of CancerGPT and other LLM-based models across different tissues and model settings. We then investigated whether the LLM’s reasoning for its prediction is valid by checking its argument with scientific literature.

### Accuracy

CancerGPT showed the highest accuracy in tissues such as the liver, soft tissue, and urinary tract. CancerGPT’s accuracy increases as the number of training shots increases (Figs. 3, 4), indicating that the information gained from a few shots of data complements the prior knowledge encoded in LLM and the information in external data. In contrast, the two data-driven models, Collaborative filtering and TabTransformer, showed higher accuracy in some tissues, including endometrium, stomach, and bone, indicating that the patterns learned from external data (common tissues) can be extrapolated to these tissues. The discrepancy in accuracy between CancerGPT (which utilizes prior knowledge and external data) and the data-driven model can be attributed to the similarity of the external data’s distribution to the data of interest. Certain rare tissue cancer cell lines show unique characteristics in comparison to common tissues. These tissues have specific cellular characteristics that are unique to their tissue of origin, which may not be accurately predicted through training with common tissue. For example, hepatic cell lines, which originate from liver tissue, have unique drug response characteristics due to high expression of drug-metabolizing enzymes such as cytochrome P450s^21^. When the data from common tissue is distributed differently to each rare tissue (i.e., external data is out-of-distribution), CancerGPT outperforms the general data-driven prediction model trained with out-of-distribution data, thanks to general prior knowledge already encoded in the LLM parameters. On the other hand, TabTransformer and Collaborative filtering, trained with external data, achieved the best accuracy in tissues such as endometrium, stomach, and bone. We further investigated the genomic characteristics of these tissues to ascertain any commonalities with common tissues. Supplementary Figure 3 showed the lower dimensional projection of gene expression of cancer cell lines grouped by tissue types^22^. In this figure, gene expression of tumorous cells from the endometrium (uterus), stomach (gastric), and bone were similar to that from other majority of tissues. Conversely, the gene expression of tumorous cells from liver, soft tissue (including Ewing sarcoma), and urinary tract formed unique clusters that were distinct from other majority of tissues. When the data from common tissues are distributed similarly to each rare tissue (in-distribution), the data-driven prediction model outperforms the few-shot LLM-based models. In all, the data-driven model showed superior accuracy when in-distribution external data was available, compared to all the LLM-based models, including CancerGPT. However, in the absence of external data, or when the external data is out-of-distribution, our customized LLM-based model achieved the best accuracy.

When comparing the accuracy of LLM-based prediction models, CancerGPT emerged as the most accurate one. This superior performance is attributable to its fine-tuning with external data, tailored specifically to the task at hand. GPT-3 also proved to be a competitive model, showing a potential to enhance its accuracy with an increasing number of shots. This characteristic renders it an optimal choice for datasets that permit the abundant additional training samples. Particularly, GPT-3 scored the highest accuracy in pancreas tissue, in which only zero-shot tuning is possible due to the limited sample size (Supplementary Tables 2 and 3). Despite SciFive’s specialized design for scientific literature and its substantial parameter size of 220M, it failed to consistently outperform GPT-2 across all datasets. The SciFive’s lower accuracy despite the larger parameters and adaptation to scientific literature might be due to its difference in base LLM (T5 and GPT). The higher accuracy of CancerGPT over GPT-2 underscores the value of task-specific adjustments. These modifications can augment accuracy while preserving a model’s versatility. However, the advantages of such fine-tuning may diminish with larger LLM models like GPT-3, particularly in scenarios demanding greater generalizability. Interestingly, CancerGPT, despite its smaller parameter size of 124M, outperformed GPT-3’s accuracy, which has a larger parameter size of 175B. This suggests that further fine-tuning of GPT-3 may potentially yield even greater accuracy, provided that in-distribution data is readily available.

We also compared the accuracy when the models are fine-tuned with different strategies. Full training, which updates both LLM parameters and classification head during k-shot tuning, generally showed higher accuracy over last layer training (Supplementary Figs. 1 and 2), which only updates the classification head while freezing the LLM parameters. However, the marginal increase in accuracy, despite the extensive tuning of the millions or even billions of parameters was not significantly high. This suggests that the last layer representation of LLM already encapsulates a substantial amount of prior knowledge, effectively serving as a foundation for downstream tasks. The observation that last layer training, which leaves the LLM’s backbone untouched, delivers accuracy comparable to that of a fully trained model, provides an important insight. An LLM pre-trained with an extensive collection of scientific literature can flexibly enhance biomedical predictions via transfer learning, which is similar to how a pre-trained model with ImageNet^23^ can augment image analysis through transfer learning.

### Fact check LLM’s reasoning

We evaluated whether the LLM can provide the biological reasoning behind its prediction. In this experiment, we used zero-shot GPT-3 because other fine-tuned LLM-based models (GPT-2, SciFive, CancerGPT) compromised its language generative performance during the fine-tuning and were not able to provide coherent responses. To do this, we randomly selected one true positive prediction and examined whether its biological rationale was based on factual evidence or mere hallucination. Our example was the drug pair AZD4877 and AZD1208 at cell line T24 for urinary tract tissue. We prompted the LLMs with *“Could you provide details on why the drug1 and drug2 are synergistic in the cell line for a given cancer type?”* Details on prompt generation are discussed in Supplementary Note 1. We evaluated the generated answer by comparing it with existing scientific literature. We found that the LLM provided mostly accurate arguments, except for two cases (Table 1) in which no scientific literature exists. By combining these individual scientific facts the LLM inferred the unseen synergistic effect. Generally, drugs targeting non-overlapping proteins in similar pathways are more likely to be synergistic^24,25^. In this case, both AZD4877 and AZD1208 target similar pathways that inhibit tumor cell divisions without overlapping protein targets. The Loewe synergy score of this pair at T24 was 46.82, meaning strong positive synergistic effect.

**Table 1:** Example of generated answer when the LLM was asked to provide its reasoning for its prediction

Excerpt of the generated answer	Fact check and reference
*“The combination of AZD-4877 and AZD1208 has been studied in T24 cells...to be synergistic in reducing bladder cancer cell growth and metastasis”*	False. No study conducted on this drug pair
*“The combination was also found to target multiple pathways involved in the growth and spread of bladder cancer cells.”*	True. AZD1208 is a PIM1 inhibitor. PIM1 is overexpressed in bladder cancer initiation and progression (^42^). AZD4877 is a drug designed to target bladder cancer (^43^).
*“...Specifically, AZD-4877 was found to inhibit the activation of proteins involved in the promotion of tumor growth...”*	True. AZD4877 is a drug designed to target bladder cancer (^43^).
*“...AZD1208 was found to inhibit proteins associated with the inhibition of tumor growth.”*	True. AZD1208 inhibits the cell growth by suppressing p70S6K, 4EBP1 phosphorylation, and messenger RNA translation (in acute myeloid leukemia) (^44^).
*“This combination was also effective at reducing the production of inflammatory mediators such as cytokines, which are known to contribute to tumor progression.”*	False. AZD1208 is a pan-PIM kinase inhibitor, and PIM kinases are downstream effectors of cytokine (^45^). However, AZD4877 has no evidence in reducing inflammatory mediators.
*“...these two drugs have been shown to reduce levels of apoptosis inhibitors, which can also play a role in tumor progression.”*	True. AZD1208 induce cell apoptosis (^46^). AZD4877 is a inhibitor of Eg5, which promotes cell apoptosis (^47^).

Italic denotes the generated text from LLM. *LLM* Large Language Model.

### Example of prediction results

As an example, we listed predicted synergistic drug pairs for stomach and soft tissue using CancerGPT (Supplementary Table 6, 7) and for bone and liver tissue using GPT-3 (Supplementary Table 8, 9). We randomly selected two true positive, false positive, true negative, and false negative prediction examples. We discovered that Loewe synergy scores of the true negative or false negative prediction examples were close to the threshold used to categorize the label (i.e., Loewe score > 5). This suggests that accuracy may vary significantly by different thresholds for determining positive synergy. Setting more extreme thresholds (e.g., >10, >30), like previous models^13,17,18^, may increase the prediction accuracy.

## Discussion

Our study investigates the potential of LLMs as a widely applicable few-shot prediction model in the field of biomedicine. Specifically, we propose a new few-shot model for predicting drug pair synergy, which can be used in rare tissues with few or no training samples available. We transformed tabular data prediction into natural language tasks and fine-tuned LLMs (GPT-2, GPT-3, SciFive) with very few samples in each tissue. Our extensive experiments with seven rare cancer tissues, seven different models, and two different finetuning strategies resulted in the following lessons learned:

- The data-driven models showed superior accuracy when in-distribution external data was available. However, in the absence of external data, or when the external data is out-of-distribution, our customized LLM-based model, CancerGPT, achieved the best accuracy.
- The higher accuracy of CancerGPT over GPT-2 underscores the value of task-specific adjustments. These modifications can augment accuracy while preserving a model’s versatility. However, the advantages of such fine-tuning may diminish with larger LLM models like GPT-3.
- CancerGPT, despite its smaller parameter size of 124M, outperformed GPT-3’s accuracy, which has a larger parameter size of 175B, suggesting that further fine-tuning of GPT-3 may potentially yield even greater accuracy, provided that in-distribution data is readily available.
- Last layer training, which leaves the LLM’s backbone untouched, delivered accuracy comparable to that of a fully trained model. An LLM pre-trained with an extensive collection of scientific literature can flexibly enhance biomedical predictions using transfer learning based on last-layer training.

The prediction of drug pair synergy in rare tissues serves as an excellent benchmark task for evaluating LLMs in few-shot learning within the field of biomedicine. This prediction requires incorporating multiple pieces of information, such as the drugs and the cell line, as well as the sensitivity of drugs to the cell lines, to infer the synergistic effects. While detailed information on these entities can be found in scientific papers, the interaction effect, or synergistic effect, is primarily available through biological experiments. To effectively assess LLMs’ inference capabilities, one must employ a prediction task where the ground truth is not explicitly available in text format but can be determined through alternative sources for model evaluation. Typically, drug pair synergy scores are obtained through high-throughput testing facilities involving robot arms^26^. Therefore, individual records of the experiments are rarely recorded in academic literature, decreasing the likelihood of their use as training data for LLMs and avoiding data leakage. Additionally, few studies have been conducted on rare tissues regarding their synergy prediction models, and their synergy prediction outcomes are not explicitly stated in text format. A similar task for evaluating LLMs in biomedicine is predicting the sensitivity of a single drug in a cell line; however, since the sensitivity of individual drugs is extensively researched and well-documented in publications, the LLM model may merely recollect from the text rather than infer unseen tasks.

It should be noted that it was not possible to compare our LLM-based models directly with previous predictions of drug pair synergy. The majority of previous models necessitates high-dimensional features of drugs and cells (e.g., genomic or chemical profiles), along with a substantial amount of training data, even the one specifically designed for rare tissue^18^. This kind of data is not easily accessible in rare tissues, making it challenging to carry out a significant comparison. Our model is designed to address a common but often overlooked situation where we have limited features and data. Thus, we compared the LLM-based models with other tabular models that share the same set of inputs.

The contribution of our study can be summarized as follows. In the area of drug pair synergy prediction in rare tissues, our study is the first to predict drug pair synergy on tissues with very limited data and features, which other previous prediction models have neglected. This breakthrough has significant implications for drug development in these cancer types. By accurately predicting which drug pair will have a synergistic effect on these tissues, where cell line features are expensive to obtain, biologists can directly focus on the most probable drug pairs and perform experiments in a cost-effective manner.

Our study also delivers generalizable insights about LLMs in the broader context of biomedicine. Our research essentially bridges two distinct methodologies: data-driven machine learning models, which rely on inductive reasoning; and knowledge-driven inference models, which use deductive reasoning. In many cases, especially when dealing with rare diseases like certain types of cancer, the data required to build data-driven models is scarce. This scarcity renders these models virtually ineffective. Conversely, knowledge-driven models pose their own challenges. Without extensive domain expertise, navigating the vast and intricate premises to predict biomedical outcomes is not only challenging, but also unscalable. Our approach combines the strengths of both models. We “automate” deductive reasoning using a LLM, which then forms the foundation for a few-shot prediction model. This combination of inductive (few-shot fine-tuning) and deductive (knowledge encapsulated in LLM) reasoning is a novel concept, made possible through our LLM-based prediction model.

Furthermore, this LLM-based few-shot prediction approach can be applied to a wide range of diseases beyond cancer, which are limited by the scarcity of available data. For instance, this approach can be used in infectious diseases, in which the prompt identification of new treatments and diagnostic tools is crucial. LLMs are able to help researchers quickly identify potential drug targets and biomarkers for these diseases, resulting in faster and more effective treatment development.

The present study, while aiming to showcase the potential of LLMs as a few-shot prediction model in the field of biomedicine, is not without its limitations. To fully establish the generalizability of LLMs as a “generalist” artificial intelligence, a wider range of biological prediction tasks must be undertaken for validation. Additionally, it is crucial to investigate how the information gleaned from LLMs complements the existing genomic or chemical features that are traditionally the primary source of predictive information. In future research, we plan to investigate this aspect and develop an ensemble method that effectively utilizes both existing structured features and new prior knowledge encoded in LLMs.

Furthermore, while we observed that GPT-3’s reasoning was similar to our own when fact-checking its argument with scientific literature in one example, it is important to note that the accuracy of its arguments cannot always be verified and may be susceptible to hallucination. It is also reported that LLMs can also contain biases that humans have^27^. Therefore, further research is necessary to ensure that the LLM’s reasoning is grounded in factual evidence. Despite these limitations, our study provides valuable insights into the potential of LLM to be a few-shot prediction model in biomedicine and lays the groundwork for future research in this area.

In conclusion, our study has effectively demonstrated the potential of LLMs in few-shot learning tasks within the complex field of biomedicine. Our study revealed that while data-driven models excel with sufficient in-distribution data, CancerGPT is superior in scenarios lacking such data. The effectiveness of task-specific fine-tuning was highlighted by the superior performance of CancerGPT over GPT-2, demonstrating that such adjustments can significantly enhance accuracy without compromising the model’s flexibility. Our findings not only are pivotal for the prediction of drug pair synergy in rare tissues but also signify broader applicability for LLMs in biological inference tasks.

## Methods

### Problem formulation

Our objective is to predict whether a drug pair in a certain cell line has a synergistic effect, particularly focusing on rare tissues with limited training samples. Given an input

1 $$x=\{{d}_{1},{d}_{2},c,t,r{i}_{1},r{i}_{2}\}$$

of drug pair (*d*_1_, *d*_2_), cell line *c*, tissue *t*, and the sensitivity of the two drugs using relative inhibition (*ri*_1_, *ri*_2_), the prediction model is

2 $$y\,\approx\, f(x)$$

where *y* is the binary synergy class (1 if synergy > 5; 0 otherwise). Prior research^14,17^ has employed three different scenarios for predicting drug pair synergy (random split, stratified by cell lines, stratified by drug combinations). Our task is to predict synergy when the data are stratified by tissue, which is a subset of cell lines.

Distributions learned in one tissue may not generalizable well to other tissues with different cellular environments^22^. This biological difference poses a challenge in predicting drug pair’s synergy in tissues with a limited number of samples. The limited sample size of data makes it even more difficult to incorporate typical cell line features, such as gene expression level, which has large dimensionality (e.g., ~ 20,000 genes). Due to this data challenge, the drug pair synergy prediction model is then reduced to build a prediction model with limited samples (few-shot or zero-shot learning) with only limited tabular input feature types. Specific input features were described in Methods.

### Prompt selection

To use a LLM for tabular data, the tabular input and prediction task must be transformed into natural text. For each instance of tabular data (Fig. 2), we converted the structured features into text. A prior study^19^ investigated different strategies to “serialize” the structured instance to natural language input, such as List Template (a list of column names and feature values), Text Template (a textual enumeration of all features and values), and Table-To-Text (table to text generation via LLM). Among them, we used the Text Template strategy which proved to be most effective. For example, given the feature string (e.g., “drug1”, “drug 2”, “cell line”, “tissue”, “sensitivity1”, “sensitivity2”) and its value (e.g., “lonidamine”, “717906-29-1”, “A-673”, “bone”, “0.568”, “28.871”), we converted the instance as *“The first drug is AZD1775. The second drug is AZACITIDINE. The cell line is SF-295. Tissue is bone. The first drug’s sensitivity using relative inhibition is 0.568. The second drug’s sensitivity using relative inhibition is 28.871.”* Other alternative ways to convert the tabular instance into the natural text are discussed in previous papers^28,29^.

We created a prompt that specifies our tasks and guides the LLM to generate a label of interest. We experimented with multiple prompts. One example of the prompts we created was “*Determine cancer drug combination synergy for the following drugs. Allowed synergies: Positive, Not positive. {Tabular Input}. Synergy:*”. As our task is a binary classification, we created the prompt to only generate binary answers (*“Positive”, “Not positive”*). Comparing these multiple prompts (Supplementary Note 1), the final prompt we used in this work was “*Decide in a single word if the synergy of the drug combination in the cell line is positive or not*. {*Tabular Input*}. *Synergy:*”.

### LLM-based prediction model

**Fig. 5:**
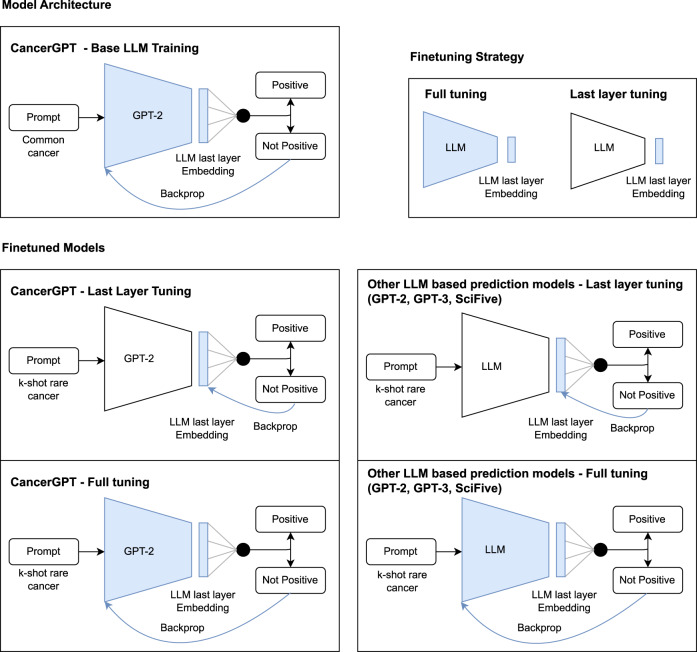
Model architecture. We built an LLM-based prediction model by adding a classification head to the LLM and fine-tuned it for the classification task. To obtain representation from LLM, we used GPT-2, GPT-3, and SciFive. We further tailored GPT-2 by fine-tuning it with a large amount of external data, in order to adjust GPT-2 in the context of drug pair synergy prediction (CancerGPT). We finetuned all models with *k* shots of data in each of the rare tissues (*k*-shot fine-tuning strategy). We compared accuracy of two different fine-tuning strategies: fine-tuning the entire model’s parameters versus freezing the LLM’s parameters and only fine-tuning the classifier’s parameters. LLM Large Language Model.

We built an LLM-based prediction model by adding a classification head to the LLM and fine-tuned it for the classification task (Fig. 5). This was done to enhance the model’s performance in the classification task. The hidden representation of the final token in the LLM output was utilized, as it encapsulates the information of all preceding tokens. This strategy not only allowed for a fair comparison with other baseline models, such as TabTransformer, but also created an opportunity to fine-tune the model specifically for binary classification.

We also considered another modeling approach, which was to allow the LLM to directly generate text for the binary response (*“positive”, “not positive”*). However, regardless of the LLM’s ability to encapsulate relevant knowledge within their embedding, some LLMs (e.g., GPT-2, SciFive) could not ensure the response strictly adhered to one of the binary labels. This made the inclusion of the classification head an essential choice. The primary objective of our study was to assess the LLM’s biomedical knowledge and its adaptability to a specific task of interest, rather than comparing language generation capabilities.

**Fig. 6:**
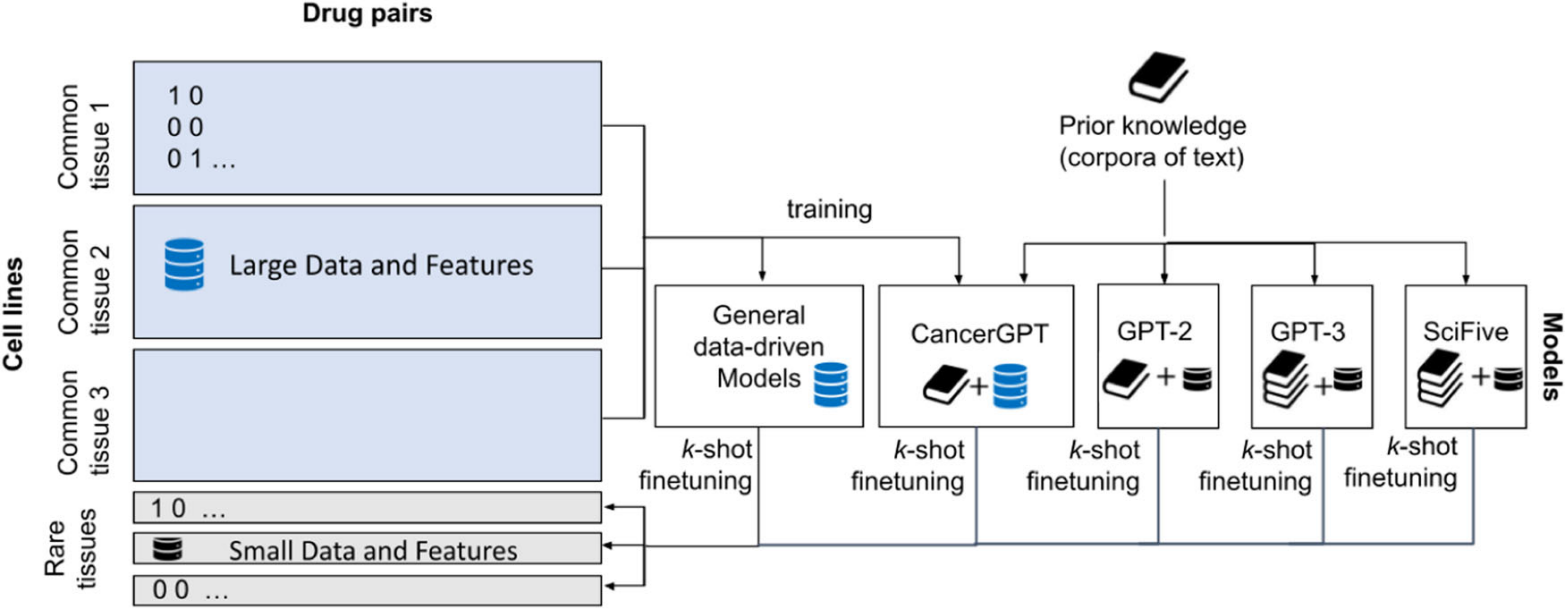
Training strategy of baseline and proposed LLM-based models. General data-driven models and CancerGPT were first trained with samples from common tissues (cancer type) then *k*-shot fine-tuned with each tissue of interest. GPT-2, GPT-3 and SciFive are pre-trained models, and we fine-tuned them with *k* shots of data in each tissue.

To obtain representation from LLM, we used GPT-2, GPT-3, and SciFive (Fig. 6). GPT-2 is a Transformer-based large language model, which was pre-trained on a very large corpus of English data without human supervision. It achieved state-of-the-art results on several language modeling datasets in a zero-shot setting when it was released, and it is the predecessor of GPT-3 and GPT-4. GPT-2^5^ has several versions with different sizes of parameters, GPT-2, GPT-Medium, GPT-Large, and GPT-XL. We used GPT-2 with the smallest number of parameters (regular GPT-2, 124 million) in this work to make the model trainable on our server.

GPT-3^2^ is a Transformer-based autoregressive language model with 175 billion parameters, and it achieved state-of-the-art performance on many zero-shot and few-shot tasks upon its release. GPT-3.5, including ChatGPT^30^, a famous fine-tuned model from GPT-3.5, is an improved version of GPT-3. However, the GPT-3 model and its parameters are not publicly available. Although the weight of the GPT-3 model is undisclosed, OpenAI offers an API^31^ to fine-tune the model and evaluate its performance. We utilized this API to build drug pair synergy prediction models through *k*-shot fine-tuning. There are four models provided by OpenAI for fine-tuning, Davinci, Curie, Babbage, and Ada, of which Ada is the fastest model and has comparable performance with larger models for classification tasks. For that reason, we use GPT-3 Ada as our classification model. After uploading the train data, the API adjusted the learning rate, which is 0.05, 0.1, or 0.2 multiplied by the original learning rate based on the size of the data, and fine-tuned the model for four epochs. A model of the last epoch was provided for further evaluation.

In contrast to general-purpose LLM (i.e., GPT-2 and GPT-3), SciFive^20^ is an LLM specialized in the biomedical field. Based on T5 model, which is a text-to-text language model trained on the unfiltered CommonCrawl dataset, SciFive re-trains the T5 model on various combinations of the CommonCrawl dataset, a corpus of PubMed abstracts, and a corpus of PubMed Central (PMC) full-text articles. We used the SciFive Pubmed Base model with 220M parameters.

### CancerGPT

We further tailored GPT-2 by fine-tuning it with a large amount of external data (common cancer data), in order to adjust GPT-2 in the context of drug pair synergy prediction. We named this model CancerGPT (Fig. 5). The data from common tissues (Data Availability)) are external data that do not necessarily align with the context of the rare tissue being studied. However, this data could potentially serve as a useful data source to “warm up” the LLM, thereby enhancing its ability to predict drug pair synergy. The intuition behind this “warming up” strategy is analogous to collaborative filtering^32^ that learn relational information and extrapolate it to the new combination of input. Certain drug pairs exhibit synergy similarly regardless of the cellular context, and therefore, the relational information between drug pairs in common tissues can be used to predict synergy in new cell lines in different tissues^17^. Subsequently, we utilized CancerGPT as one of the pre-trained LLMs and fine-tuned to *k* shots of data in each rare tissue (as discussed in the following section).

### General data-driven models

The LLM-based prediction are knowledge-driven models as they utilize prior knowledge encoded in pre-trained weight. We compared them with general data-driven prediction model. We specifically used XGBoost^33^, TabTransformer^34^, and collaborative filtering^18^. XGBoost has been widely used in large-scale drug synergy data^35,36^. All the variables (drugs, cell lines, and sensitivities) were used as input to predict the drug pair synergy. TabTransformer is a self-attention-based supervised learning model for tabular data. TabTransformer applies a sequence of multi-head attention-based Transformer layers on parametric embedding to transform them into contextual embedding, in which highly correlated features will be close to each other in the embedding space. Considering the highly correlated nature of drugs in our data, TabTransformer can be a very strong baseline in this work. We first converted the drugs and cell lines in the tabular data into indicators using one-hot coding. We first trained an embedding layer on the drugs and cell lines and passed them through stacked multi-headed attention layers, which we then combined with the continuous variables (sensitivities). This combination then passes through feed-forward layers, which have one layer of classification head. Collaborative filtering was used to extrapolate the relational information to the new combination of drugs and cell line. Drug combinations will react to a cell line similarly if these two drugs have responded similarly to other cell line^18^. Note that, tissue information was not used in training because the models will be tested in one specific rare tissue that is not used in training. No further contextual information can be inferred through the unseen tissue indicator.

### *k*-shot fine-tuning strategy

The LLM-based models had different training and fine-tuning strategies (Fig. 6). Samples of common tissues were split into 80% train data and 20% validation data for CancerGPT. The models were trained using train data and evaluated by validation data to determine the models with specific hyperparameters to be used for further fine-tuning on rare tissues. For the GPT-2 and GPT-3 based prediction models, we directly used pre-trained parameters from GPT-2^5^ using Huggingface’s Transformers library^37^ and GPT-3 Ada from OpenAI^2^ respectively.

All these models were then fine-tuned with *k* shots of data in each of the rare tissues. For bone, urinary tract, stomach, soft tissue, and liver, we performed experiments with *k* from [0, 2, 4, 8, 16, 32, 64, 128]. For endometrium and pancreas, because of the limited number of data, we implemented experiments with *k* from [0, 2, 4, 8, 16, 32] from the endometrium, and only zero shot (*k* = 0) for the pancreas.

With the limited number of shots, a careful balance of binary labels in the train and test set was critical. We partitioned the data into 80% for training and 20% for testing in each rare tissue, while ensuring the binary labels were equally represented in both sets. We randomly selected *k* shots from the training for fine-tuning, while maintaining consistency with previously selected shots and adding new ones. Specifically, we maintained the previously selected *k* shots in the training set and incremented additional *k* shots to create 2 × *k* shots. The binary label distribution in each *k* shot set followed that of the original data, with at least one positive and one negative sample included in each set. For evaluation stability, the test data was consistent across different shots for each tissue.

To investigate whether the LLMs inherently possesses the capability to predict the label only with minimal tuning, we compared accuracy of two different fine-tuning strategies: fine-tuning the entire model (LLM’s parameter and classifier) versus freezing the LLM’s parameter and fine-tuning only on the classifier. Note that for zero shot tuning, we measured accuracy by allowing the LLM to directly generate a binary answer without appending a classification head because we are unable to optimize the parameters in the classification head.

### Hyperparameter setting

The LLM’s last layer hidden representation size was 768 (same as GPT-2), and we used left padding to ensure that the last token was from the prompt sentence. The cross-entropy loss was used to optimize the model during the fine-tuning process. All the LLM models used the tabular input that was converted to natural text and shared the same prompt.

The predicted output was a binary label indicating the presence of a synergistic effect, with a Loewe score greater than 5 indicating a positive result. We used AUROC and AUPRC to evaluate the accuracy of classification. Regression tasks were not possible in our LLM-based models because our model can only generate text-based answers (*“positive”* or *“not positive”*), with poor precision in accurately quantifying the synergy value.

XGBoost was used with a boosting learning rate of 0.3. The number of gradient boost trees was set to 1000 with a maximum tree depth of 20 for base learners. TabTransformer was used with a learning rate of 0.0001 and a weight decay of 0.01. The model was trained for 50 epochs on common tissues. During the training, the model with the best validation performance was selected for further fine-tuned on rare tissues. For each *k* shot in each tissue, the model was fine-tuned using the same learning rate and weight decay for 1 epoch and tested with AUPRC and AUROC.

CancerGPT was first fine-tuned with pre-trained regular GPT-2 for 4 epochs on common tissues. The learning rate was set to be 5e-5 and weight decay was set to be 0.01. Then the model was fine-tuned for *k* shots in rare tissues. The same hyperparameters are used in training. The model was finally tested with AUPRC and AUROC. Note that, due to an imbalance in positive and non-positive labels, we reported both AUPRC and AUROC.

GPT-2 and GPT-3 are directly fine-tuned on rare tissues with pre-trained parameters from regular GPT-2 and GPT-3 Ada. For each *k* shot in each tissue, GPT-2 is fine-tuned for 4 epochs using a learning rate of 5e-5 and a weight decay of 0.01. The hyperparameters of GPT-3 are adjusted by OpenAI API based on the data size. The model was also fine-tuned for 4 epochs. GPT-2, GPT-3 and SciFive fine-tuned models were finally tested with AUPRC and AUROC. Details in the hyperparameter setting are discussed in Supplementary Note 2.

### Reporting summary

Further information on research design is available in the Nature Research Reporting Summary linked to this article.

### Supplementary information

CancerGPT - Supplementary

Reporting Summary

## Data Availability

We utilized a publicly accessible extensive database of drug synergy from DrugComb Portal^9^, which is an open-access data portal where the results of drug combination screening studies for a large variety of cancer cell lines are accumulated, standardized, and harmonized. The database contains both drug sensitivity rows and drug pair synergy rows. After filtering the available drug pair synergy rows, the data contains 4226 unique drugs, 288 cell lines, with a total of 718,002 drug pair synergy rows. We employed the Loewe synergy score, which ranges from -100 (antagonistic effect) to 75 (strong synergistic effect), for drug combination synergy^38^. The Loewe synergy score quantifies the excess over the expected response if the two drugs are the same compound^39,40^. In this paper, we focused on cell lines from rare tissues. We defined the rare tissues as the ones with less than 4000 samples, which include the pancreas (*n* = 39), endometrium (*n* = 68), liver (*n* = 213), soft tissue (*n* = 352), stomach (*n* = 1190), urinary tract (*n* = 2458), and bone (*n* = 3985). We tested our models with each of the rare tissues. The remaining tissues, namely hematopoietic and lymphoid, lung, skin, ovary, kidney, colon, brain, breast, and prostate were regarded as common tissues.

## Code availability

All models used in this paper were developed using open-sourced libraries such as PyTorch^41^, HuggingFace^37^ and publically available APIs from OpenAI^31^. Method-specific code will be provided upon request.
