# Supplementary material for: CancerGPT for few shot drug pair synergy prediction using large pretrained language models
Source: NPJ Digit Med. 2024 Feb 19;7:40. doi: 10.1038/s41746-024-01024-9 (PMC10876664; doi:10.1038/s41746-024-01024-9)
Supplement: Supplementary file 1 — CancerGPT - Supplementary [file 41746_2024_1024_MOESM1_ESM.pdf]

Supplementary materials

Supplementary Note 1: Prompt engineering

Prompts is one of the few variables we can control manually while using pre-trained LLMs (especially with zero-shot). We tried a variety of prompts to stimulate our models to provide the best output for both classification and to provide a reasonable explanation for the results. We share below some of the prompts tried on zero-shot GPT-3.

For classification it is important to specify the output classes that we are expecting the model to choose amongst. In its absence, the model tends to generate longer sequences by rephrasing the question before concluding with the answer that we expect.

Prompt: Determine the drug synergy combination for the given cancer drug combination. The first drug is *CHIR-99021*. The second drug is *SF1670*. Cell line is *AGS*. Tissue is stomach. Synergy:
Output: "The combination of CHIR-99021 and SF1670 has been studied … suggests that this drug combination could be a potential therapeutic option for gastric cancer."

On the other hand, manually specifying the class list makes the model generate the output we expect as the first word itself.
Prompt: Determine the drug synergy combination for the given cancer drug combination. Allowed synergies: {positive, not positive}. The first drug is CHIR-99021. The second drug is SF1670. Cell line is AGS. Tissue is stomach. Synergy:
Output: "Positive."

For reasoning, correct prompts can determine the level of details we receive from the model. We show two slightly different prompts, one produces a generic answer (below), while the other is more specific to the drugs
Prompt: Why are drugs AZD-4877 and AZD1208 synergistic in cell line T24 for urinary_tract cancer?
Reasoning: "…The combination of these two drugs has been shown to significantly reduce tumor growth and cell proliferation ... target different pathways involved in the growth and metastasis of the cancer …"

Prompt: Could you explain in detail why drugs AZD-4877 and AZD1208 are synergistic in cell line T24 for urinary_tract cancer?
Reasoning: **"**The combination of AZD-4877 and AZD1208 has been studied in T24 cells...to be synergistic in reducing bladder cancer cell growth and metastasis … Specifically, AZD-4877 was found to inhibit the activation of proteins involved in the promotion of tumor growth.. ..AZD1208 was found to inhibit proteins associated with the inhibition of tumor growth … effective at reducing the production of inflammatory mediators such as cytokines, which are known to contribute to tumor progression … reduce levels of apoptosis inhibitors, which can also play a role in tumor progression"

We go over in detail and perform a fact-checking analysis of GPT-3's response in Fact check LLM's reasoning. Not only was it able to provide direct facts about the drugs, but also return some implied information mentioned in a related paper about a different type of cancer [1], [2]. However, some facts were not found out to not be true.

Supplementary Note 2: Hyperparameter summary

Fine-tuning	TabTransformer	GPT2	CancerGPT	GPT3
Epoch	1	4	4	4
Learning Rate	0.0001	5e-5	5e-5	API adjusted
Weight Decay	0.01	0.01	0.01	API adjusted
Loss Function	Cross-entropy	Cross-entropy	Cross-entropy	Cross-entropy
Optimizer	AdamW	AdamW	AdamW	AdamW

Supplementary Table 1: Hyperparameter summary

Supplementary Note 3: Ablation Study

	Methods	Number of Shots							
		0	2	4	8	16	32	64	128
Pancreas (n_0=38,n_1=1)	XGBoost	0.5	-	-	-	-	-	-	-
	Collaborative Filtering	0.763	-	-	-	-	-	-	-
	TabTransformer	0.211	-	-	-	-	-	-	-
	CancerGPT	0.132	-	-	-	-	-	-	-
	GPT-2	0.316	-	-	-	-	-	-	-
	SciFive	0.342	-	-	-	-	-	-	-
	GPT-3	**0.789**	-	-	-	-	-	-	-
Liver (n_0=192,n_1=21)	XGBoost	0.587	0.587	0.587	0.587	0.587	0.587	0.574	0.574
	Collaborative Filtering	0.635	0.705	0.65	0.564	0.667	0.538	0.692	0.75
	TabTransformer	0.76	0.753	0.76	0.747	0.837	0.824	0.76	0.74
	CancerGPT	**0.853**	**0.795**	**0.833**	**0.827**	**0.962**	**0.948**	**0.84**	0.833
	GPT-2	0.66	0.468	0.532	0.442	0.647	0.75	0.635	0.59
	SciFive	0.603	0.609	0.641	0.5	0.872	0.801	0.596	0.73
	GPT-3	0.615	0.49	0.542	0.583	0.474	0.731	0.737	**0.91**
Soft Tissue (n_0=269,n_1=83)	XGBoost	0.491	0.491	0.491	0.491	0.454	0.476	0.542	0.552
	Collaborative Filtering	0.582	0.677	0.589	0.6	0.672	0.679	0.66	0.637
	TabTransformer	0.399	0.299	0.332	0.459	0.72	0.79	0.781	0.756
	CancerGPT	**0.805**	**0.75**	**0.803**	**0.827**	**0.817**	**0.871**	**0.899**	0.9
	GPT-2	0.398	0.344	0.465	0.279	0.459	0.302	0.394	0.506
	SciFive	0.412	0.467	0.421	0.64	0.541	0.589	0.82	**0.901**
	GPT-3	0.517	0.406	0.6	0.444	0.607	0.82	0.866	0.889
Urinary Tract (n_0=1996,n_1=462)	XGBoost	0.494	0.494	0.494	0.494	0.494	0.526	0.53	0.544
	Collaborative Filtering	0.612	**0.628**	**0.653**	0.595	0.609	0.597	0.62	0.629
	TabTransformer	0.493	0.483	0.482	0.499	0.48	0.498	0.501	0.492
	CancerGPT	0.611	**0.595**	**0.604**	**0.605**	**0.625**	**0.624**	**0.63**	**0.653**
	GPT-2	0.477	0.378	0.475	0.421	0.474	0.453	0.482	0.47
	SciFive	0.612	0.532	0.55	0.562	0.486	0.477	0.551	0.528
	GPT-3	**0.645**	0.57	0.556	0.496	0.508	0.516	0.531	0.572
Endometrium (n_0=36,n_1=32)	XGBoost	0.5	0.5	0.5	0.5	0.5	0.5	-	-
	Collaborative Filtering	**0.878**	0.694	0.673	0.755	0.693	0.796	-	-
	TabTransformer	0.327	0.571	0.816	**0.939**	**0.939**	**0.918**	-	-
	CancerGPT	0.551	0.571	0.571	0.571	0.673	0.714	-	-
	GPT-2	0.163	0.816	0.878	0.653	0.612	0.776	-	-
	SciFive	0.449	0.429	0.612	0.592	0.347	0.755		
	GPT-3	**0.837**	**1**	**0.949**	0.898	0.878	0.898	-	-
Stomach (n_0=1081,n_1=109)	XGBoost	0.529	0.529	0.529	0.529	0.529	0.529	0.476	0.508
	Collaborative Filtering	0.771	0.79	0.784	0.79	0.76	0.799	0.788	0.768
	TabTransformer	0.731	**0.865**	**0.851**	0.796	0.724	0.75	0.785	0.781
	CancerGPT	**0.799**	0.804	0.816	**0.822**	**0.822**	**0.821**	**0.831**	**0.796**
	GPT-2	0.607	0.579	0.481	0.579	0.519	0.543	0.428	0.424
	SciFive	0.541	0.588	0.527	0.637	0.582	0.458	0.622	0.515
	GPT-3	0.419	0.575	0.724	0.769	0.534	0.69	0.742	0.724
Bone (n_0=3732,n_1=253)	XGBoost	0.499	0.499	0.499	0.499	0.499	0.499	0.499	0.499
	Collaborative Filtering	**0.782**	**0.762**	**0.78**	**0.77**	**0.78**	**0.752**	**0.772**	0.774
	TabTransformer	0.547	0.587	**0.69**	**0.706**	**0.696**	**0.729**	**0.728**	0.746
	CancerGPT	0.57	**0.613**	0.645	0.6	0.59	0.625	0.574	0.668
	GPT-2	0.487	0.566	0.471	0.647	0.587	0.421	0.623	0.586
	SciFive	**0.599**	0.443	0.645	0.551	0.596	0.528	0.622	0.65
	GPT-3	0.498	0.415	0.341	0.429	0.485	0.605	0.62	**0.794**

Supplementary Table 2: AUROC Full Tuning

n_0=total number of non-synergistic samples (not positive), n_1=total number of synergistic samples (positive)

	Methods	Number of Shots							
		0	2	4	8	16	32	64	128
Pancreas	XGBoost	0.026	-	-	-	-	-	-	-
	Collaborative Filtering	0.1	-	-	-	-	-	-	-
	TabTransformer	0.032	-	-	-	-	-	-	-
	CancerGPT	0.032	-	-	-	-	-	-	-
	GPT-2	0.037	-	-	-	-	-	-	-
	SciFive	0.038	-	-	-	-	-	-	-
	GPT-3	**0.111**	-	-	-	-	-	-	-
Liver	XGBoost	0.132	0.132	0.132	0.132	0.132	0.132	0.12	0.12
	Collaborative Filtering	0.355	**0.388**	**0.373**	0.341	0.236	0.208	0.216	0.407
	TabTransformer	0.228	**0.284**	**0.286**	**0.404**	0.532	0.326	0.218	0.191
	CancerGPT	0.294	0.24	0.264	0.257	**0.729**	**0.642**	**0.45**	0.446
	GPT-2	0.156	0.102	0.148	0.101	0.353	0.417	0.209	0.178
	SciFive	**0.378**	0.168	0.172	0.112	0.362	0.439	0.22	0.382
	GPT-3	0.185	0.086	0.096	0.125	0.124	0.314	0.362	**0.519**
Soft Tissue	XGBoost	0.243	0.243	0.243	0.243	0.235	0.235	0.264	0.271
	Collaborative Filtering	0.3	0.34	0.321	0.359	0.367	0.431	0.354	0.369
	TabTransformer	0.263	0.197	0.213	0.259	0.485	0.558	0.594	0.525
	CancerGPT	**0.473**	**0.398**	**0.461**	**0.519**	**0.521**	**0.682**	**0.703**	0.599
	GPT-2	0.261	0.188	0.296	0.173	0.221	0.203	0.267	0.332
	SciFive	0.208	0.316	0.242	0.353	0.269	0.346	0.631	**0.767**
	GPT-3	0.263	0.194	0.28	0.228	0.363	0.618	0.638	0.734
Urinary Tract	XGBoost	0.186	0.186	0.186	0.186	0.186	0.197	0.199	0.209
	Collaborative Filtering	0.255	**0.273**	0.266	0.261	0.243	0.256	0.274	0.267
	TabTransformer	0.187	0.194	0.194	0.194	0.182	0.189	0.189	0.182
	CancerGPT	0.264	**0.263**	**0.287**	**0.278**	**0.302**	**0.303**	**0.31**	**0.324**
	GPT-2	0.172	0.145	0.17	0.175	0.176	0.181	0.172	0.17
	SciFive	0.254	0.194	0.208	0.233	0.179	0.174	0.205	0.211
	GPT-3	**0.27**	0.228	0.222	0.201	0.206	0.2	0.24	0.272
Endometrium	XGBoost	0.5	0.5	0.5	0.5	0.5	0.5	-	-
	Collaborative Filtering	**0.904**	0.794	0.687	0.755	0.654	0.862	-	-
	TabTransformer	0.459	0.632	0.86	**0.948**	**0.948**	**0.933**	-	-
	CancerGPT	0.513	0.583	0.539	0.536	0.639	0.525	-	-
	GPT-2	0.379	0.821	0.86	0.723	0.779	0.82	-	-
	SciFive	0.479	0.474	0.605	0.738	0.799	0.674	-	-
	GPT-3	**0.869**	**1**	**0.947**	0.859	0.799	0.859	-	-
Stomach	XGBoost	0.104	0.104	0.104	0.104	0.104	0.104	0.09	0.094
	Collaborative Filtering	0.352	**0.437**	**0.374**	**0.404**	0.338	**0.443**	**0.459**	0.35
	TabTransformer	0.214	**0.408**	**0.355**	0.293	0.24	0.259	0.298	0.341
	CancerGPT	**0.352**	0.331	0.342	**0.335**	**0.41**	**0.43**	**0.419**	**0.389**
	GPT-2	0.123	0.115	0.143	0.111	0.098	0.108	0.08	0.083
	SciFive	0.104	0.134	0.107	0.216	0.145	0.084	0.152	0.129
	GPT-3	0.078	0.106	0.17	0.37	0.1	0.19	0.219	0.181
Bone	XGBoost	0.064	0.064	0.064	0.064	0.064	0.064	0.064	0.064
	Collaborative Filtering	**0.174**	**0.147**	**0.176**	**0.163**	**0.172**	0.164	**0.186**	0.165
	TabTransformer	0.09	**0.11**	**0.139**	**0.152**	**0.142**	**0.166**	**0.158**	0.176
	CancerGPT	0.091	0.101	0.112	0.09	0.095	0.101	0.076	0.12
	GPT-2	0.065	0.079	0.069	0.097	0.085	0.062	0.086	0.087
	SciFive	**0.103**	0.067	0.116	0.087	0.09	0.081	0.088	0.097
	GPT-3	0.064	0.051	0.045	0.058	0.068	0.087	0.101	**0.181**

Supplementary Table 3: AUPRC Full Tuning

	Methods	Number of Shots						
		2	4	8	16	32	64	128
Liver	TabTransformer	**0.686**	**0.801**	**0.788**	0.827	**0.859**	**0.788**	0.84
	CancerGPT	0.474	0.603	0.66	**0.853**	0.814	0.737	**0.904**
	GPT-2	0.365	0.686	0.66	0.801	0.801	0.75	0.891
	SciFive	0.609	0.692	0.686	0.731	0.699	0.66	0.724
	GPT-3	0.417	0.506	0.442	0.878	0.795	0.731	0.776
Soft Tissue	TabTransformer	0.138	0.242	0.463	0.598	0.668	0.818	0.82
	CancerGPT	**0.618**	0.632	0.684	0.703	0.751	0.777	0.867
	GPT-2	0.467	**0.671**	0.619	0.604	**0.901**	**0.938**	0.862
	SciFive	0.594	0.604	**0.699**	**0.758**	0.788	0.847	0.821
	GPT-3	0.492	0.667	0.624	0.734	0.888	0.869	**0.878**
Urinary Tract	TabTransformer	0.501	0.491	0.523	0.456	0.512	0.566	0.585
	CancerGPT	0.563	0.458	0.425	0.608	0.546	0.625	0.645
	GPT-2	0.552	0.499	0.515	0.518	0.553	0.571	0.648
	SciFive	**0.629**	**0.659**	**0.661**	**0.655**	**0.651**	**0.654**	**0.668**
	GPT-3	0.62	0.6	0.604	0.575	0.539	0.609	0.634
Endometrium	TabTransformer	**0.918**	**0.959**	0.918	0.959	0.878	-	-
	CancerGPT	0.265	0.265	0.531	0.551	0.816	-	-
	GPT-2	0.469	0.714	**1**	**1**	**1**	-	-
	SciFive	0.673	0.633	0.857	0.878	0.857	-	-
	GPT-3	0.347	0.592	0.694	0.673	0.857	-	-
Stomach	TabTransformer	0.585	0.529	0.639	0.626	0.581	0.659	**0.813**
	CancerGPT	0.767	**0.785**	**0.777**	0.714	0.734	0.751	0.691
	GPT-2	0.743	0.617	0.724	**0.73**	**0.757**	0.721	0.7
	SciFive	0.48	0.452	0.515	0.545	0.517	0.593	0.6
	GPT-3	**0.774**	0.756	0.714	0.698	0.732	**0.782**	0.731
Bone	TabTransformer	**0.717**	**0.786**	**0.776**	**0.757**	**0.71**	0.677	0.732
	CancerGPT	0.651	0.623	0.612	0.601	0.609	0.613	0.68
	GPT-2	0.373	0.434	0.522	0.607	0.67	0.627	**0.734**
	SciFive	0.396	0.596	0.622	0.584	0.496	0.493	0.565
	GPT-3	0.657	0.678	0.688	0.69	0.697	**0.688**	0.721

Supplementary Table 4: AUROC Last Layer Tuning

	Methods	Number of Shots						
		2	4	8	16	32	64	128
Liver	TabTransformer	**0.243**	**0.516**	**0.48**	**0.55**	0.3	0.233	0.352
	CancerGPT	0.12	0.132	0.238	0.343	**0.599**	**0.392**	**0.674**
	GPT-2	0.091	0.371	0.377	0.523	0.44	0.257	0.421
	SciFive	0.186	0.313	0.296	0.346	0.448	0.377	0.449
	GPT-3	0.126	0.137	0.123	0.405	0.469	0.38	0.401
Soft Tissue	TabTransformer	0.151	0.171	0.249	0.315	0.354	0.646	0.61
	CancerGPT	0.298	0.304	0.349	0.364	0.41	0.431	0.575
	GPT-2	0.276	0.404	0.337	0.355	**0.708**	**0.833**	**0.73**
	SciFive	**0.543**	**0.469**	**0.519**	**0.53**	0.462	0.531	0.504
	GPT-3	0.263	0.379	0.337	0.468	0.662	0.609	0.662
Urinary Tract	TabTransformer	0.253	0.219	0.237	0.188	0.21	0.228	0.248
	CancerGPT	0.232	0.187	0.162	0.266	0.218	0.282	0.287
	GPT-2	0.217	0.188	0.213	0.229	0.231	0.267	0.315
	SciFive	**0.279**	**0.305**	**0.333**	**0.324**	**0.31**	**0.332**	**0.35**
	GPT-3	0.3	0.279	0.268	0.25	0.232	0.319	0.284
Endometrium	TabTransformer	**0.933**	**0.962**	0.909	0.962	0.826	-	-
	CancerGPT	0.427	0.42	0.613	0.562	0.833	-	-
	GPT-2	0.537	0.633	**1**	**1**	**1**	-	-
	SciFive	0.687	0.659	0.886	0.91	0.881	-	-
	GPT-3	0.433	0.569	0.662	0.653	0.844	-	-
Stomach	TabTransformer	0.159	0.134	0.185	0.181	0.204	0.233	0.373
	CancerGPT	0.239	0.31	**0.301**	0.285	**0.309**	**0.401**	**0.418**
	GPT-2	0.281	0.164	0.295	**0.287**	0.256	0.316	0.197
	SciFive	0.129	0.102	0.097	0.104	0.099	0.117	0.142
	GPT-3	**0.318**	**0.314**	0.255	0.227	0.253	0.272	0.201
Bone	TabTransformer	**0.142**	**0.154**	**0.182**	**0.168**	**0.18**	**0.164**	**0.197**
	CancerGPT	0.119	0.111	0.107	0.1	0.119	0.097	0.136
	GPT-2	0.05	0.07	0.079	0.094	0.12	0.127	0.168
	SciFive	0.059	0.089	0.1	0.089	0.073	0.076	0.086
	GPT-3	0.102	0.105	0.107	0.108	0.107	0.11	0.153

Supplementary Table 5: AUPRC Last Layer Tuning

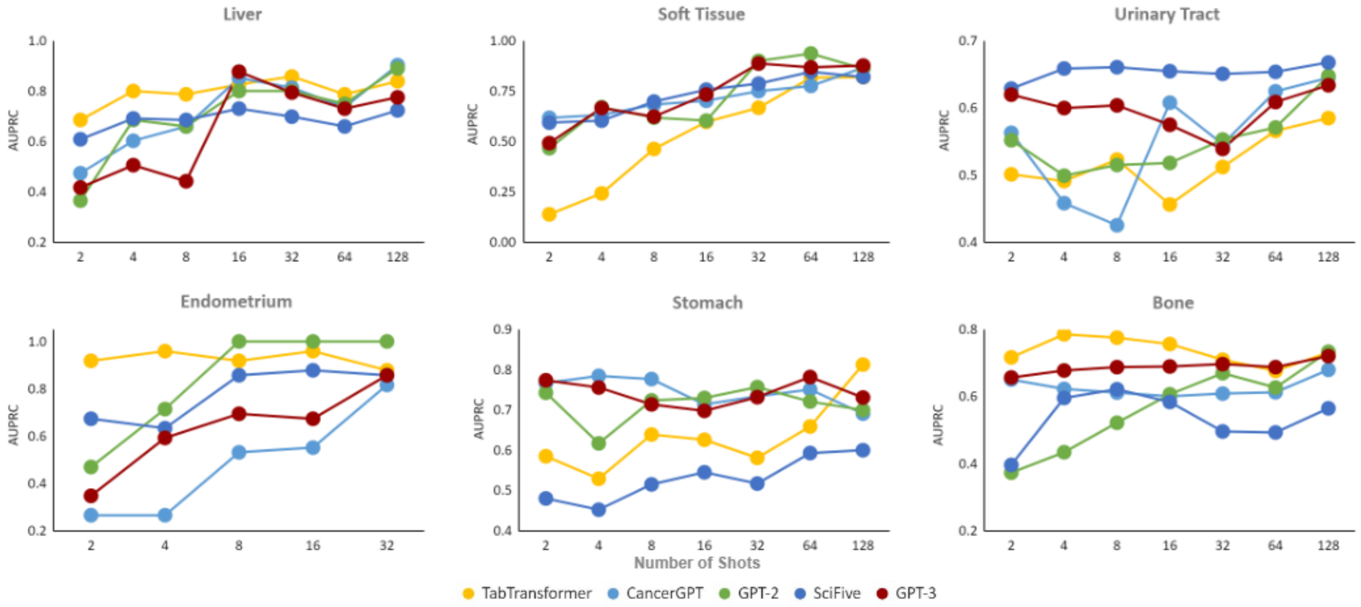

Yellow: TabTransformer, Light Blue: CancerGPT, Green: GPT-2, Dark Blue: SciFive, Red: GPT-3
Supplementary Figure 1: AUROC of k-shot learning on seven tissue sets with finetuning only the last layer

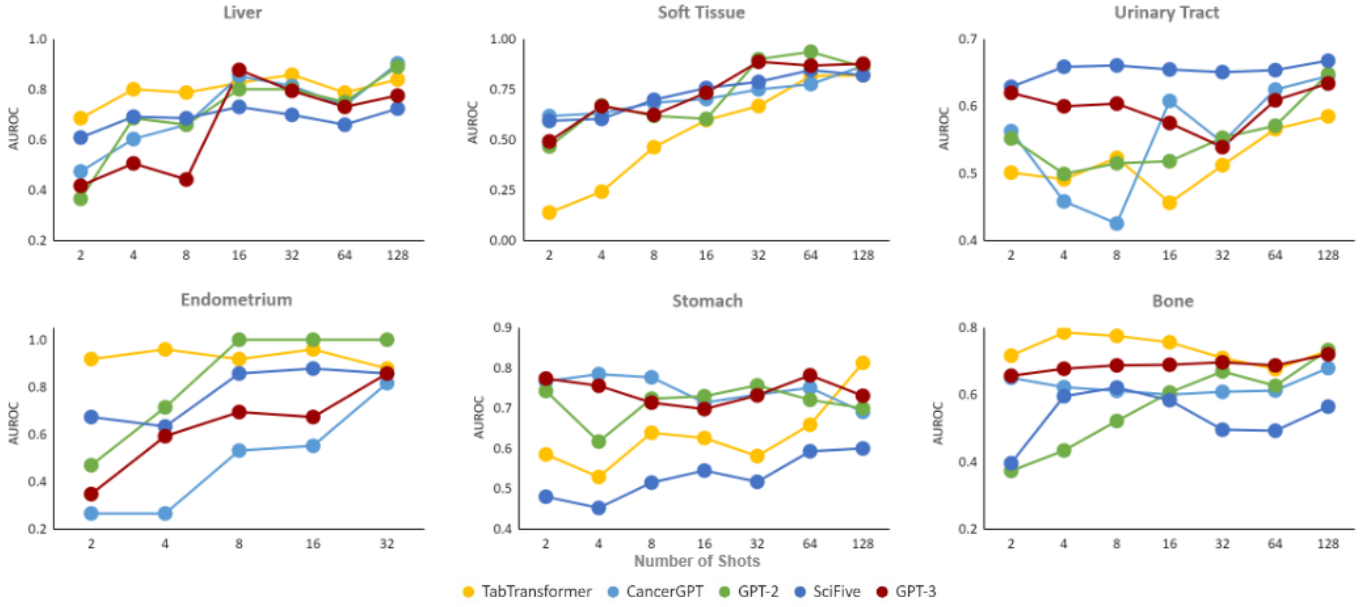

Yellow: TabTransformer, Light Blue: CancerGPT, Green: GPT-2, Dark Blue: SciFive, Red: GPT-3
Supplementary Figure 2: AUPRC of k-shot learning on seven tissue sets with finetuning only the last layer

Supplementary Note 4: Prediction Examples

	Predicted	
Ground truth	Synergy	No Synergy
Synergy	(Akt inhibitor VIII, Doramapimod, AGS)<br>(Doramapimod, SF1670, AGS)	(5Z-7-Oxozeaenol, 10058-F4, AGS)<br>(5Z-7-Oxozeaenol, BI605906, AGS)
No Synergy	(Akt inhibitor VIII, SF1670, AGS)<br>(PI-103, SF1670, AGS)	(CHIR-99021, GSK429286A, AGS)<br>(BI605906, 10058-F4, AGS)

Supplementary Table 6: Example of prediction in stomach tissue (Drug A, Drug B, Cell line)

	Predicted	
Ground truth	Synergy	No Synergy
Synergy	(915019-65-7, Trametinib, SMS-CTR)<br>(Omipalisib, Trametinib, RD)	(Omipalisib, ceritinib, RD)<br>(SCHEMBL82368, Niraparib, Rh36)
No Synergy	(BMS-754807, MK-2206, SMS-CTR)<br>(MK-1775, 955-24-8, Rh36)	(Stattic, SU11274, DDLS8817)<br>(Akt inhibitor VIII, Stattic, DDLS8817)

Supplementary Table 7: Example of prediction in soft tissue (Drug A, Drug B, Cell line)

	Predicted	
Ground truth	Synergy	No Synergy
Synergy	(Romidepsin, Thapsigargin, EW-8)<br>(Daporinad, Panobinostat, TC-32)	(Carfilzomib, Entinostat, TC-32)<br>(Mocetinostat, Carfilzomib, TC-71)
No Synergy	(Thapsigargin, Beta-Narcotine, TC-32)<br>(SCHEMBL82368, Olaparib, TC-71)	(Ethyl bromopyruvate, 717906-29-1, A-673)<br>(Doxorubicin Hydrochloride, Olaparib, TC-71)

Supplementary Table 8: Example of prediction in bone tissue (Drug A, Drug B, Cell line)

		Predicted	
		Synergy	No Synergy
Ground truth	Synergy	(Piperacetazine, Aripiprazole, Huh-7)<br>(Idrocilamide, Sertraline hydrochloride, Huh-7)	(3-Deazaneplanocin, Piperacetazine, Huh-7)<br>(Bepridil, Sertraline hydrochloride, Huh-7)
	No Synergy	(Toremifene Citrate, Aripiprazole, Huh-7)<br>(Idrocilamide, Clomifene citrate, Huh-7)	(Clomiphene, Mycophenolate Mofetil, Huh-7)<br>(3-Deazaneplanocin, Colchicine, Huh-7)

Supplementary Table 9: Example of prediction in liver tissue (Drug A, Drug B, Cell line)

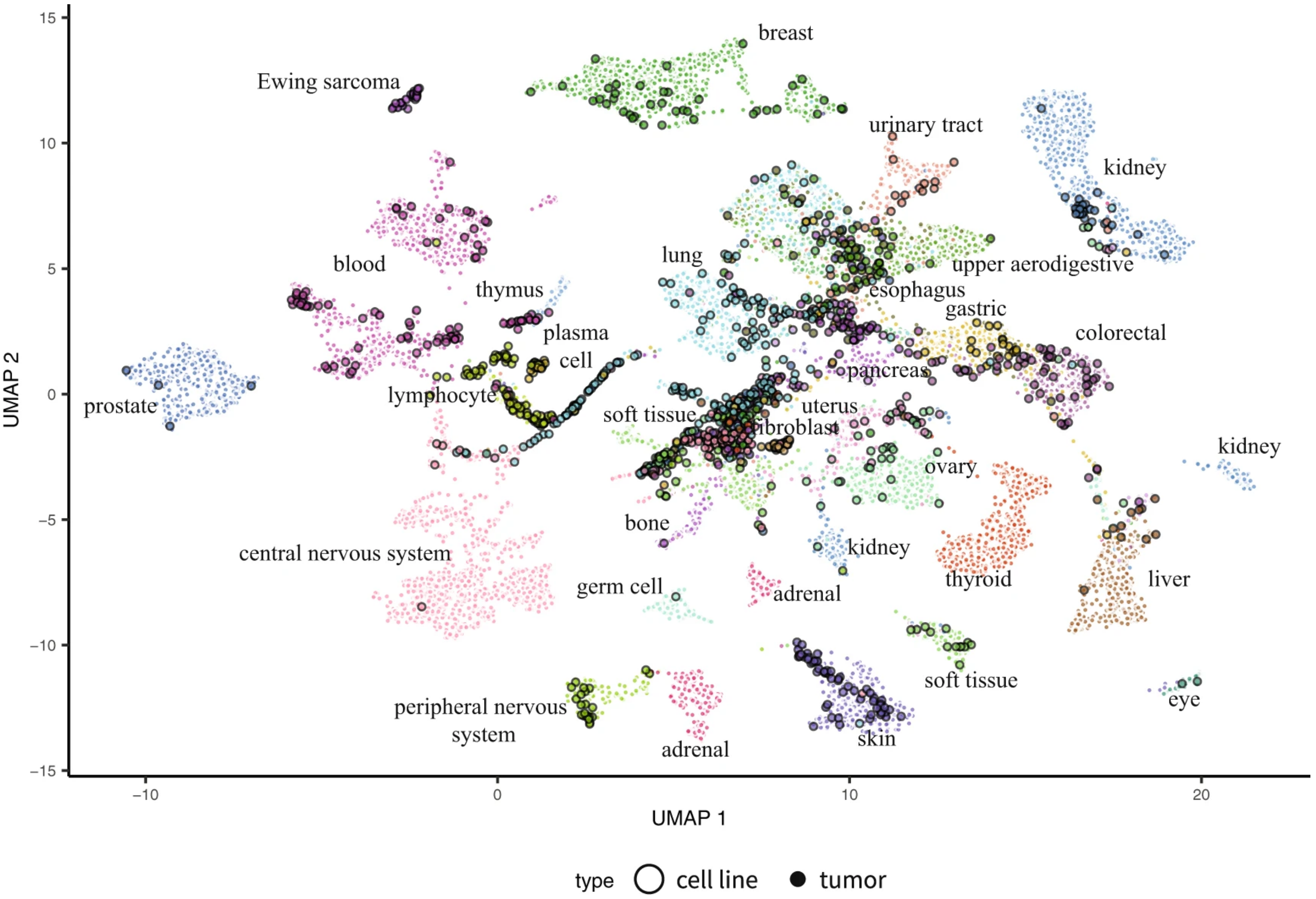

Supplementary Figure 3: UMAP 2D projection of Celligner- aligned tumor and cell line expression data colored by annotated cancer lineage. The alignment includes 12,236 tumor samples and 1,249 cell lines, across 37 cancer types. This figure is adapted from [3]

Supplementary References

1. Yadav, A. K., Kumar, V., Bailey, D. B. & Jang, B.-C. AZD1208, a Pan-Pim Kinase Inhibitor, Has Anti-Growth Effect on 93T449 Human Liposarcoma Cells via Control of the Expression and Phosphorylation of Pim-3, mTOR, 4EBP-1, S6, STAT-3 and AMPK. *Int. J. Mol. Sci.* **20**, (2019).
2. Guo, S. *et al.* Overexpression of Pim-1 in bladder cancer. *J. Exp. Clin. Cancer Res.* **29**, 161 (2010).
3. Warren, A. *et al.* Global computational alignment of tumor and cell line transcriptional profiles. *Nat. Commun.* **12**, 22 (2021).
